# PNIPAM Brushes in Colloidal Photonic Crystals Enable Ex Situ Ethanol Vapor Sensing

**DOI:** 10.1021/acsapm.3c02397

**Published:** 2023-12-14

**Authors:** Esli Diepenbroek, Maria Brió Pérez, Sissi de Beer

**Affiliations:** Department of Molecules & Materials, MESA+ Institute, University of Twente, 7522 NB Enschede, The Netherlands

**Keywords:** structural colors, nanoparticles, polymer brushes, vapor sensing, smart materials

## Abstract

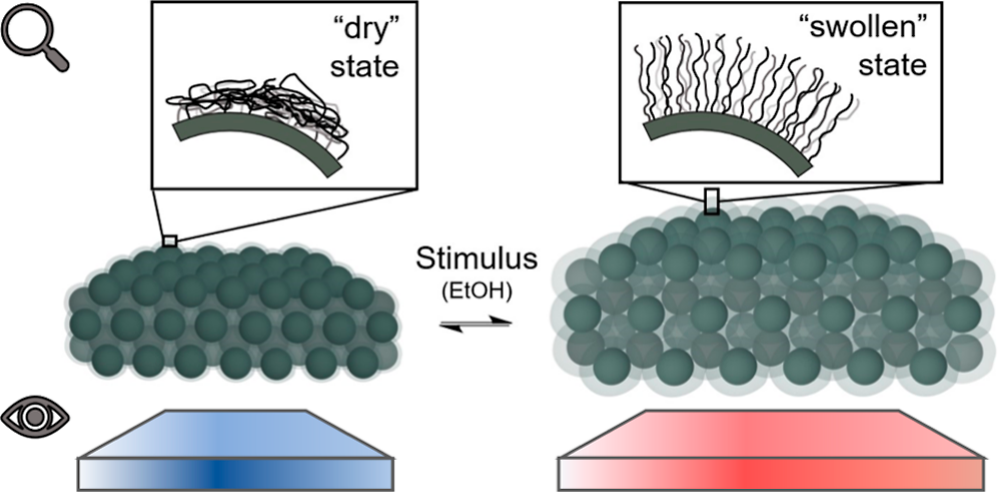

Structural colors are formed by the periodic repetition of nanostructures in a material. Upon reversibly tuning the size or optical properties of the repetitive unit inside a nanostructured material, responsive materials can be made that change color due to external stimuli. This paper presents a simple method to obtain films of ethanol vapor-responsive structural colors based on stacked poly(*N*-isopropylacrylamide) (PNIPAM)-grafted silica nanoparticles. Our materials show clear, reversible color transitions in the presence of near-saturated ethanol vapor. Moreover, due to the absorption of ethanol in the PNIPAM brushes, relatively long recovery times are observed (∼30 s). Materials based on bare or poly(methyl methacrylate) (PMMA) brush-grafted silica nanoparticles also change color in the presence of ethanol vapor but possess significantly shorter recovery times (∼1 s). Atomic force microscopy reveals that the delayed recovery originates from the ability of PNIPAM brushes to swell in ethanol vapor. This renders the films highly suitable for ex situ ethanol vapor sensing.

## 1 Introduction

Colors are typically used to accentuate, mask, signal, or simply differentiate between different objects.^1^ Herein, bright and vivid colors originate from either chromophores or nanosized repetitive structures within the material itself.^1−3^ The latter depicts the category of “structural colors”.^1,4,5^

Structurally colored materials are able to interfere with, diffract, or scatter light of a specific wavelength in the visible light range (370–700 nm).^1,6^ These materials have attracted a lot of attention because they do not require dye-based constituents, maintain their vivid and bright color longer,^5^ and can be tuned such that they show these colors in a one-, two- or three-dimensional manner.^4,7,8^ Examples of these respective configurations are so-called “Bragg–Stacks” (1D), diffraction gratings (2D), and colloidal photonic crystals (3D).^7−9^ Due to their versatility, cost-effectiveness, and easy preparation, colloids such as silica, latex, and polystyrene (PS) nanoparticles have been assembled into colloidal photonic crystals that display opal effects.^3,10−12^

If the dimensions and optical properties of the repetitive unit within structural colors are changed in a reversible way, then the materials exhibit stimuli-responsive behavior. In the literature, successful attempts were reported of structurally colored materials that respond to pH,^4^ humidity,^1,13,14^ temperature,^4^ UV light,^6^ mechanical stress,^8^ magnetic fields,^15^ applied voltages,^16^ or the presence of biomolecules.^17^

Herein, the nanostructures that were used to induce the required structural changes include etalons,^4^ nanovolcanos,^18^ hydrogels,^1,13,14,19^ thin films,^6^ magnetic nanoparticles,^15^ catalytically active nanopillars,^16^ and polymer brush-grafted nanoparticles.^17^

Stimuli-responsive structural colors have been used in vapor sensors due to their nonfatigue properties.^20,21^ Examples of such materials are humidity and organic vapor sensors based on chameleon-inspired actuators^20^ and Bragg reflectors.^19,21,22^ Due to the type of nanostructures used, all of the aforementioned materials possess a fast response and recovery time (<1–10 s).^20−22^ Fast response times are generally advantageous for sensing applications.^23^ However, while fast recovery times are essential for in situ vapor sensing, they pose a limitation for vapor sensing ex situ. Ex situ sensors are typically applied in environments that are hazardous or sterile and where in situ, real-time monitoring by colorimetric sensors is impossible. In the literature, they have been explored for temperature,^24^ water activity,^25^ and bioprocess monitoring.^26^

In recent years, polymer brushes have been heavily investigated for their stimuli-responsive features.^27−29^ Polymer brushes consist of macromolecular chains anchored by one chain end to a substrate at a sufficiently high density.^30,31^ Various polymer brush types have successfully displayed stimuli-responsive features for pH,^4,32^ humid air,^4,33^ temperature,^4,34,35^ as well as specific solvents and volatile organic compounds (VOCs).^36−38^ Based on the affinity of the polymer with its surroundings, the polymer chains will stretch away from a surface and swell in height. Herein, the swelling behavior of a polymer brush is typically expressed in the swelling ratio α.^37^ Typical swelling ratio values for polymer brushes depict 4.0–4.5 for exposure to good solvents and 1.3–2.2 for their corresponding near-saturated vapors.^36,37,39^ This difference can be explained by chemical potential difference Δμ, which is smaller in a polymer brush-vapor system compared to a polymer brush-liquid system.^29^ Additionally, typical response and recovery times are in the order of minutes,^36^ which makes polymer brushes suitable for both in situ and ex situ vapor sensing.

Polymer brushes have been used in structurally colored materials via Ag-coated nanovolcano arrays^18^ and Au-coated etalons.^4^ Herein, the polymer brushes were utilized for their responsive behavior toward water vapor, pH, and temperature.^4,18^ However, both materials require the additional use of precious metals (Ag and Au) and availability of dedicated fabrication technologies such as thermal evaporation^4,18^ and reactive ion etching (RIE).^18^

Polymer brush-grafted nanoparticles have been used as a repetitive unit in structural colors due to the self-arranging,^5^ self-healing,^40^ and stimuli-responsive^41^ properties enabled by the polymer brush coating. Thermo- and magnetically responsive structural colors were successfully fabricated based on poly(*N*-isopropylacrylamide) (PNIPAM)^41^ and poly(methyl methacrylate) (PMMA)^15^ brush-grafted nanoparticles, respectively. Solvated polymer brush systems were used in both cases to achieve noticeable structural changes and thus a color transition of the material. To the best of our knowledge, stimuli-responsive structural colors based on polymer brush-grafted nanoparticles have not yet been demonstrated in air. Therefore, it is not known whether the reduced swelling of polymer brush-vapor systems leads to noticeable, reversible color shifts in structurally colored materials. It is also undetermined whether the change in polymer brush geometry from a flat substrate to a high-surface area material affects its response and recovery times with a vapor stimulus. Studying the combination of a nanoporous structure with stimuli-responsive polymer brushes in vapor conditions may reveal different response and recovery behavior compared to conventional vapor-responsive polymer brushes or structural colors, which allow for better in situ or even ex situ vapor sensing.

**Figure 1:**
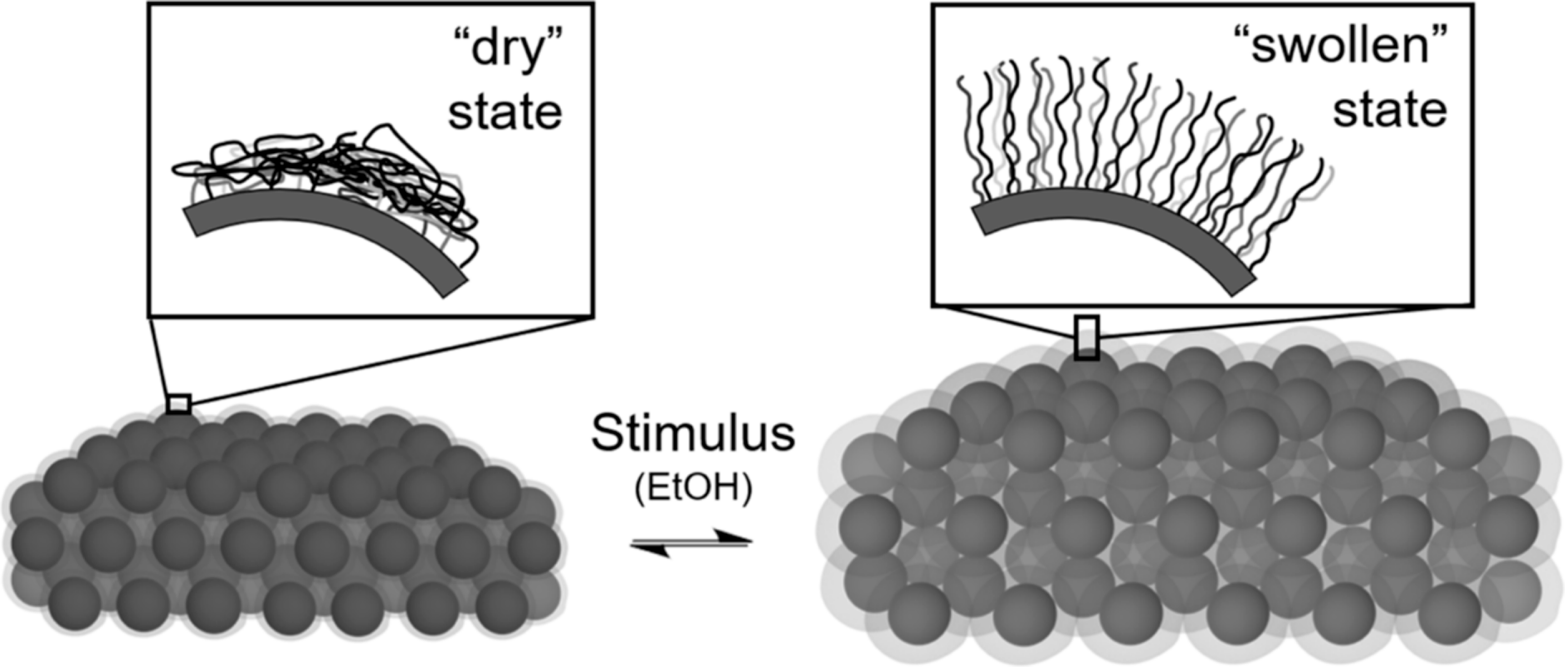
Illustration of the stacked PNIPAM-*g*-SiNPs materials and their stimuli-responsive behavior in ethanol (EtOH) vapor. In dry conditions (left), the polymer chains remain collapsed on the nanoparticle surface. In the presence of ethanol vapor (right), the chains stretch away from the surface in a “swollen” configuration. The resulting increase in nanostructural dimensions leads to a visible color change in the material.

This paper presents a facile method to obtain ethanol vapor-responsive structural colors based on orderly stacked polymer brush-grafted nanoparticles. Various films composed of PNIPAM brush-grafted silica nanoparticles (PNIPAM-*g*-SiNPs) were exposed to near-saturated ethanol vapor to study their stimuli-responsive behavior. PNIPAM is a polymer that has a satisfactory affinity to ethanol vapor^42^ and opens the possibility for future studies on multiresponsive materials due to its thermosensitivity. The novelty of our work is related to the long color recovery times found in such materials, which makes it possible for them to be used for different applications. An illustration of the material design and ethanol vapor-responsive behavior is shown in Figure 1.

The recovery behavior of the PNIPAM-*g*-SiNP films was determined to test their potential as an ex situ vapor sensor. These films were compared to reference materials with nonfunctionalized (SiNPs) and PMMA brush-grafted silica nanoparticles (PMMA-*g*-SiNPs), which have a significantly lower affinity to ethanol liquid and vapor.^43^ Structural changes of the repetitive unit were monitored before, during, and after exposure to saturated ethanol vapor to mark differences between stacked nanoparticle films with varying thicknesses and surface functionalities.

## 2 Experimental Section

### 2.1 Materials

Methyl methacrylate (MMA, 99%) was separated from its polymerization inhibitor content by an alumina oxide column. *N*-Isopropylacrylamide (NIPAM, ≥99%) was purified by heating (40 °C) and recrystallization (0 °C) in toluene. Copper^(I)^ bromide (CuBr) was cleaned with acetic acid and subsequently washed with ethanol prior to use.

Copper^(II)^ bromide (CuBr_2_, 99%), (3-aminopropyl) triethoxysilane (APTES, 99%), α-bromoisobutyryl bromide (BiBB, 98%), *N*,*N*,*N*′,*N*″,*N*‴-penta-methyldiethylenetriamine (PMDETA, 99%), triethylamine (TEA, 99%), tetraethyl orthosilicate (TEOS, ≥99%), hydrogen chloride (HCl, 60%), and ammonia (NH_4_OH, 32%) were obtained from Sigma-Aldrich and used without purification. Milli-Q water was purified from a Milli-Q Advantage A10 purification system (Millipore).

### 2.2 Stöber Protocol for 125 nm SiNPs

This Stöber protocol is adapted from Yu et al.^44^ 100 mL of ethanol, 35 mL of Milli-Q water, and 3.25 mL of NH_4_OH were placed in a 250 mL flask and heated to 65 °C. While stirring at 550 rpm, 8.0 mL of TEOS was added at 0.5 mL/s. The reaction mixture was continuously stirred for 1 h. The synthesized SiNPs were separated from the reactants by centrifugation for 30 min at 10,000 rpm (20 °C). Two washing steps in ethanol were done to obtain a stock dispersion of SiNPs in ethanol.

### 2.3 Nanoparticle Surface Preparation for SI-ATRP^45−47^

The SiNP surface functionalization steps, including the SiNP surface preparation and polymerization of PNIPAM and PMMA, are illustrated in the Supporting Information, Scheme S1.

A hydrolysis step was done to maximize the amount of OH-groups on the nanoparticle surfaces.^45^ For this reaction, a SiNP dispersion in ethanol/water (v/v ratio 204:6) was prepared. HCl was added dropwise until a solution at pH 1.0 was reached. The reaction mixture was continuously stirred at 500 rpm for 16 h, followed by centrifugation. The hydrolyzed nanoparticles were washed twice with an ethanol solvent.

Next, the SiNP surface was modified with APTES, which served as an anchoring layer. A 250 mL round-bottom flask was filled with a 4 g/100 mL dispersion of hydrolyzed SiNPs in ethanol and 2 mL of APTES. This mixture was stirred for 3 h at 700 rpm (20 °C). Subsequently, the APTES-functionalized SiNPs were collected by centrifugation. One washing step was performed with ethanol.

We are aware that APTES and other silane anchors can degraft from the substrate upon long-term exposure to organic media,^48^ ethanol/water mixtures,^49^ or humid vapor.^33^ However, the samples described in this paper were freshly prepared and not exposed to solvents for >2 days. For such immersion times, no degrafting of APTES was observed.^33^ If longer utilization times are needed, we recommend stabilizing the anchoring layer with a diblock copolymer brush with an additional hydrophobic block or a multivalent bond anchor, e.g., poly(glycidyl methacrylate).^50^

The APTES-functionalized SiNPs were solvent-exchanged to dimethylformamide (DMF) by means of centrifugation (30 min, 20 °C, 10,000 rpm). A 4 g/100 mL dispersion in DMF was cooled to 0 °C before adding 3 mL of TEA and 1 mL of BiBB dropwise and simultaneously to the flask. The reaction proceeded for 15 h at 550 rpm (20 °C). BiBB-functionalized nanoparticles were collected by using centrifugation, followed by two washing steps in DMF.

### 2.4 Preparation of PNIPAM-*g*-SiNPs

This synthesis route is adapted from Manivannan et al.^41^ PNIPAM brush growth via SI-ATRP proceeded with 180 mg of BiBB-functionalized SiNPs, 1.0 g of NIPAM, 124 μL of PMDETA, 20 mg of CuBr, 4 mL of Milli-Q, and 4 mL of methanol. The ATRP reaction flasks were stirred at 500 rpm and purged with nitrogen at 1 mL/min. After initiation of the SI-ATRP reaction, the reaction flask was continuously stirred for 1–3 h to yield different polymer brush thicknesses. The reaction was quenched upon opening the flask and centrifuging the reaction mixture. Two subsequent washing steps were carried out with water and ethanol to remove the catalyst and ligand in solution.

### 2.5 Preparation of PMMA-*g*-SiNPs^47^

PMMA brush growth via SI-ATRP proceeded with 1000 mg of BiBB-functionalized SiNPs, 4.0 mL of MMA, 94.6 μL of PMDETA, 0.0456 g of CuBr, 0.0303 g of CuBr_2_, and 43 mL of DMF. The reaction flasks were stirred at 500 rpm and purged with nitrogen at 1 mL/min. After initiation of the SI-ATRP reaction, the reaction flask was continuously stirred for 0.5–2.0 h at 65 °C to yield different polymer brush thicknesses. The reaction was quenched upon opening the flask and centrifuging the reaction mixture. Two subsequent washing steps were carried out with DMF to remove the catalyst and ligand in solution.

### 2.6 Preparation of Stacked Nanoparticle Films

#### 2.6.1 Method 1

3 × 1 cm^2^ silicon substrates were positioned in an upright position inside a snap-cap vial filled with a 1–5 wt % dispersion of PNIPAM-*g*-SiNPs in ethanol or PMMA-*g*-SiNPs in DCM. The solvent was allowed to evaporate for 2 days before removing the nanoparticle films from the vial.

#### 2.6.2 Method 2

A step motor (DC motor 23.112–050, Maxon) was used to move 1 × 1 cm^2^ silicon substrates at a constant withdrawal speed. 5 wt % dispersions of PNIPAM-*g*-SiNPs and PMMA-*g*-SiNPs were prepared in ethanol and DCM, respectively. During the dip-coating procedure, silicon substrates were immersed and withdrawn from the dispersion at a speed of 0.10–1.00 mm/s to create nanoparticle films of different thicknesses.

### 2.7 Characterization

Nanoparticle diameters were determined with scanning electron microscopy (SEM, JSM-6010LA, JEOL) and dynamic light scattering (DLS, Zetasizer Nano-ZS, Malvern Panalytical). To provide solid evidence for a polymer brush layer surrounding the SiNPs, transmission electron microscopy (TEM, Spectra300, Thermo Scientific) and Fourier transform infrared (FTIR) spectroscopy (Alpha II, Bruker) were done as well.

The ordered stacking of core–shell nanoparticles leads to the formation of structural colors, which have been characterized by cross-sectional SEM (JSM-6010LA, JEOL), reflection spectroscopy in the wavelength range of 400–700 nm (HR4000, Ocean Insights), and atomic force microscopy (AFM) (Multimode, Bruker). The AFM tapping mode was used with silicon cantilevers (NanoWorld NCH) of radius <8 nm, stiffness ∼42 N/m, and a resonance frequency of 320 kHz.

The influence of near-saturated ethanol vapor on the structure of the nanoparticle films was tested by surrounding the AFM device with a 5 L closed chamber. Inside the closed chamber, open vials with a cumulative total of 160 mL of ethanol were placed. The solvent was allowed to evaporate for at least 30 min before the first AFM images were taken. This waiting time was determined to be sufficient, as a stable and equilibrated state was observed after 30 min. The swelling ratio of PNIPAM brushes in exposure to ethanol vapor was determined with ellipsometry measurements (M2000-X, J.A. Woollam Co. Inc.).

## 3 Results and Discussion

### 3.1 General Material Characteristics

The desired polymer brush-grafted SiNPs were successfully synthesized as described in the Experimental Section. The SiNP diameter was measured to be 125.5 ± 3.2 nm by DLS and SEM (see Figure S1). PNIPAM brushes of varying dry heights (8–50 nm) were grafted from the synthesized SiNPs, as confirmed by both SEM and TEM imaging (see Table S1, Figures S1 and S2). Further details regarding the FTIR spectra and swelling characteristics of PNIPAM-*g*-SiNPs and PMMA-*g*-SiNPs can be found in the Supporting Information, Figure S3, Tables S1 and S2.

**Figure 2:**
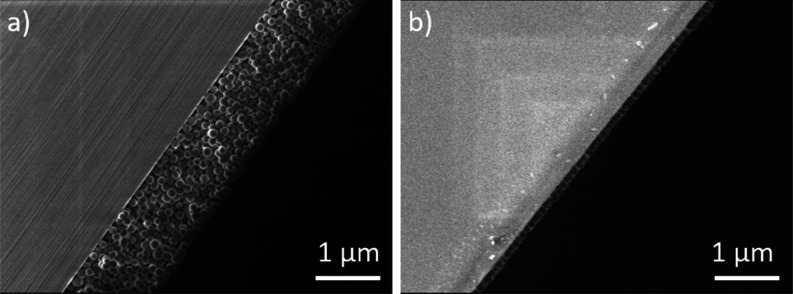
(a) SEM image of a 1.1 ± 0.01 μm thick film of stacked PNIPAM-*g*-SiNPs, obtained via method 1. (b) SEM image of a 140 ± 3 nm thick film of stacked PNIPAM-*g*-SiNPs, obtained via method 2. Both images (a,b) were taken at a 1.5 kV acceleration voltage and 20,000× magnification.

Various thicknesses of stacked PNIPAM-*g*-SiNP films were obtained via methods 1 and 2. An overview of the material characteristics is shown in Table 1, which includes the type of core–shell nanoparticle, deposition method, film thickness *t*, nanoparticle diameter *d*, and color appearance in ethanol vapor and air. Method 1 yielded relatively thick (approximately 700–1200 nm) materials with a low surface roughness (Figure 2a). Method 2 allowed for a more flexible approach, whereby monolayers and bilayers of approximately 150–230 nm in height were obtained for withdrawal speeds of 0.1–0.5 mm/s (Figure 2b). Again, the surface appeared to be devoid of protrusions that could negatively affect its optical properties. The nanoparticles within the films were closely packed (see Figure S4), which is advantageous for a structural color.

**Table 1:** Overview of the Material and Ethanol Vapor-Responsive Properties of Stacked Nanoparticles Films^a^

sample type	method	*t* (nm)	*d* (nm)	color (air)	color (EtOH)	delay (s)	relaxation (s)
PNIPAM-*g*-SiNP	1	1123 ± 21	174.6 ± 3.3	blue	pink	5.4 ± 2.8	34.0 ± 7.1
PNIPAM-*g*-SiNP	1	1152 ± 12	223.5 ± 5.4	green	pink	5.6 ± 1.1	36.2 ± 13.1
PNIPAM-*g*-SiNP	1	783 ± 8	223.5 ± 5.4	green	yellow	6.3 ± 1.5	33.3 ± 7.9
PNIPAM-*g*-SiNP	2	138 ± 2	138.0 ± 2.1	blue	yellow	6.3 ± 0.5	N/A
PMMA-*g*-SiNP	1	826 ± 7	157.7 ± 2.7	blue	green	N/A	1.5 ± 0.9
PMMA-*g*-SINP	2	162 ± 4	157.7 ± 2.7	blue	blue	N/A	N/A
SiNP	1	648 ± 8	125.5 ± 3.2	blue	yellow	N/A	0.4 ± 0.1
SiNP	2	126 ± 3	125.5 ± 3.2	blue	blue	N/A	N/A

a Information on the materials include (f.l.t.r.) the nanoparticle type, fabrication method, film thickness (*t*), nanoparticle diameter (*d*), color in air and ethanol vapor (EtOH), delay time until the initial color shift (delay), and total recovery time until the original color is regained (relaxation).

**Figure 3:**
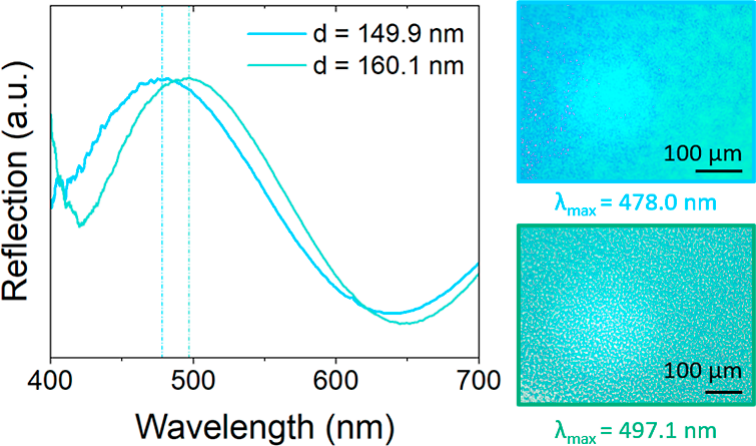
Reflection spectra of two stacked PNIPAM-*g*-SiNP films matched with images of the investigated materials. The samples are color-coded based on their sample color. The reflection peak λ_max_ of a material with smaller PNIPAM-*g*-SiNPs (149.9 nm) is positioned at a lower wavelength compared to those of larger PNIPAM-*g*-SiNPs (160.1 nm).

The resulting color of the film was dependent on the PNIPAM-*g*-SiNP diameter and angle of perception, while independent of the film thickness. As provided in Table 1, PNIPAM-*g*-SiNP diameters of 223.5 ± 5.4 nm gave rise to green structurally colored films in dry conditions, whereas blue alternatives were obtained with PNIPAM-*g*-SiNPs of 138.0 ± 2.1 nm or 174.6 ± 3.3 nm. Meanwhile, materials with the same PNIPAM-*g*-SiNP diameter of 223.5 ± 5.4 nm and different film thicknesses of 783 ± 8 and 1152 ± 12 nm possessed an identical green hue. Reflection spectroscopy revealed that an increase in the average PNIPAM-*g*-SiNP diameter also corresponded to a red shift in the reflection peak λ_max_. Figure 3 shows that an increase in average PNIPAM-*g*-SiNP diameter from 149.9 to 160.1 nm resulted in a reflection peak shift from 478.0 to 497.1 nm, respectively.

These results are in line with the theoretical expression of Bragg–Snell’s law, which describes 3D colloidal photonic crystals
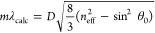
1wherein *m* is the diffraction order, λ is the reflective wavelength in nm, *D* is the colloidal spacing in nm, *n*_eff_ is the effective refractive index, and θ_0_ is the angle of incident light in degrees (^°^).^8,9,51^ For the PNIPAM-*g*-SiNP sample with λ_max_ = 492.5 nm, eq 1 predicts λ_calc_ to be 507.2 nm, assuming a FCC colloidal packing and measuring angle of 0° (see Figure S5). The difference between the theoretical prediction and experimental observation can be attributed to the influence of *n*_eff_ and *D* on the calculation of λ_max_. Both values were estimated based on the results from DLS, SEM, and AFM, and slight alterations in their values can correspond to a ±12 nm difference in λ_calc_ per parameter. This parameter influence study is included in the Supporting Information, Table S3.

Upon establishing that these stacked PNIPAM-*g*-SiNP films possess structural coloration, we studied their stimuli-responsive behavior in saturated ethanol vapor.

### 3.2 Ethanol Vapor-Responsive Material Properties

**Figure 4:**
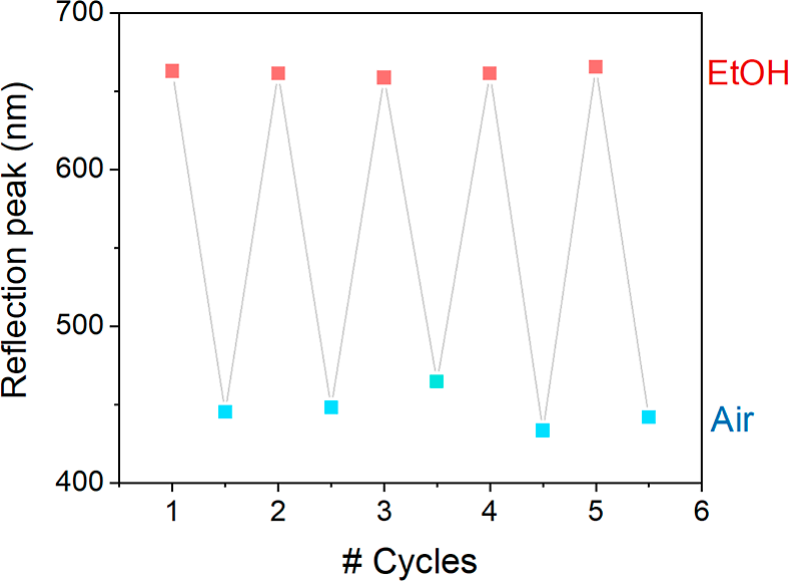
Optical stability of a PNIPAM-*g*-SiNP structural color after repeated ethanol exposure. The reflection peak wavelengths in ethanol vapor (red) and in air (blue) are plotted against the number of exposures (# cycles).

Color shifts at near-saturated and lower ethanol vapor concentrations were observed by exposing our materials to a gentle ethanol vapor flow. The samples were positioned at varying distances from this flow to qualitatively assess the sensitivity to ethanol vapor. A comparison between PNIPAM-*g*-SiNP (1) and PNIPAM-*g*-SiNP (2) films is provided in the Supporting Information (Figure S6). The stimuli-responsive behavior of various PNIPAM-*g*-SiNP films was monitored via a sealed chamber filled with ethanol vapor at near saturation level. By placing the structurally colored samples inside these sealed chambers, color changes could be observed through the transparent glass material. Due to the obstruction of the glass material, the response time of the materials in exposure to ethanol vapor could not be determined quantitatively. However, a clear red-shift color change could be seen from outside the sealed chamber in mere seconds. Despite a fast responsive behavior, our samples were kept in the sealed chamber for ∼1 day to allow for possible polymer brush swelling and equilibration. If the sealed chamber was opened after a few seconds, the samples appeared to be nonequilibrated as the recovery time increased for increasing incubation times. Therefore, the total recovery time could be more precisely determined upon opening the sealed chamber after equilibration, after which the samples were filmed. The average duration of these color transitions was determined by repeated cycles of opening and closing the sealed chamber (>5 repetitions). All color transitions were reversible and consistent throughout the repeat experiment. In addition, the color transitions of a single sample were reproducible over >5 ethanol vapor exposures (Figure 4) and at least 250 days after their fabrication (see Supporting Information, Table S4), indicating optical stability. An overview of the total recovery times of different PNIPAM-*g*-SiNP films and reference materials consisting of PMMA-*g*-SiNPs and nonfunctionalized SiNPs is shown in Table 1.

**Figure 5:**
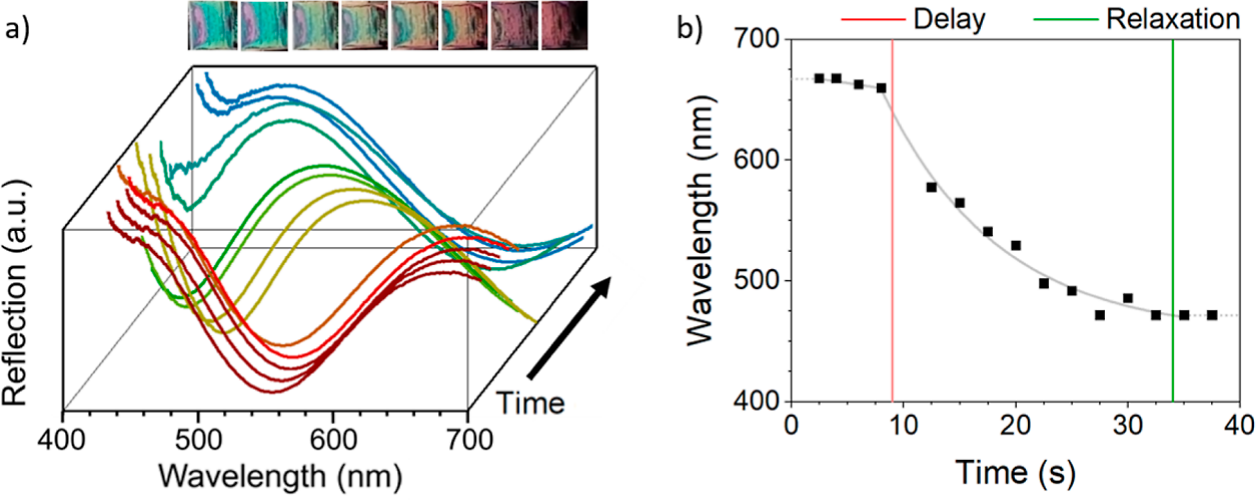
Continuous reflection spectroscopy experiment of a 1.152 ± 0.012 μm PNIPAM-*g*-SiNP film over time. Photos of the material color transition during the experiment (Δ*t* = 5 s) are accompanying (a). The reflection peak wavelengths from the individual reflection spectra (a) were plotted as data points (■) against the time axis to monitor the color transition (b). A gray line is added to guide the eye. The approximate delay (red) and relaxation (green) times are added as vertical guidelines.

Depending on the fabrication method and sample type, a wide variety of blue-shift changes were observed in the recovery phase. Interestingly, multilayered PNIPAM-*g*-SiNP(1) films were the only material type to possess a color transition between the two phases. First, a subtle shift in color was observed 5.4–6.3 s after opening the sealed chamber (see: delay). The color transition to its original state in air took significantly longer and was determined to be 33.3–36.2 s (see: relaxation). These longer recovery times closely resemble the behavior of hydrogel-based colorimetric sensors, which also report recovery times in the order of minutes.^52,53^ The delayed recovery phenomenon is more closely investigated with a continuous reflection spectroscopy experiment and is presented in Figure 5. In this experiment, multiple reflection spectra of the same material were taken over time. The corresponding reflection peaks were extracted and plotted as a function of time, which clearly illustrated the two-phase recovery behavior. Namely, a stagnant period (delay) is followed by a rapid blue shift from 667 to 577 nm, after which the reflection peak value slowly equilibrates to 471 nm (relaxation).

**Figure 6:**
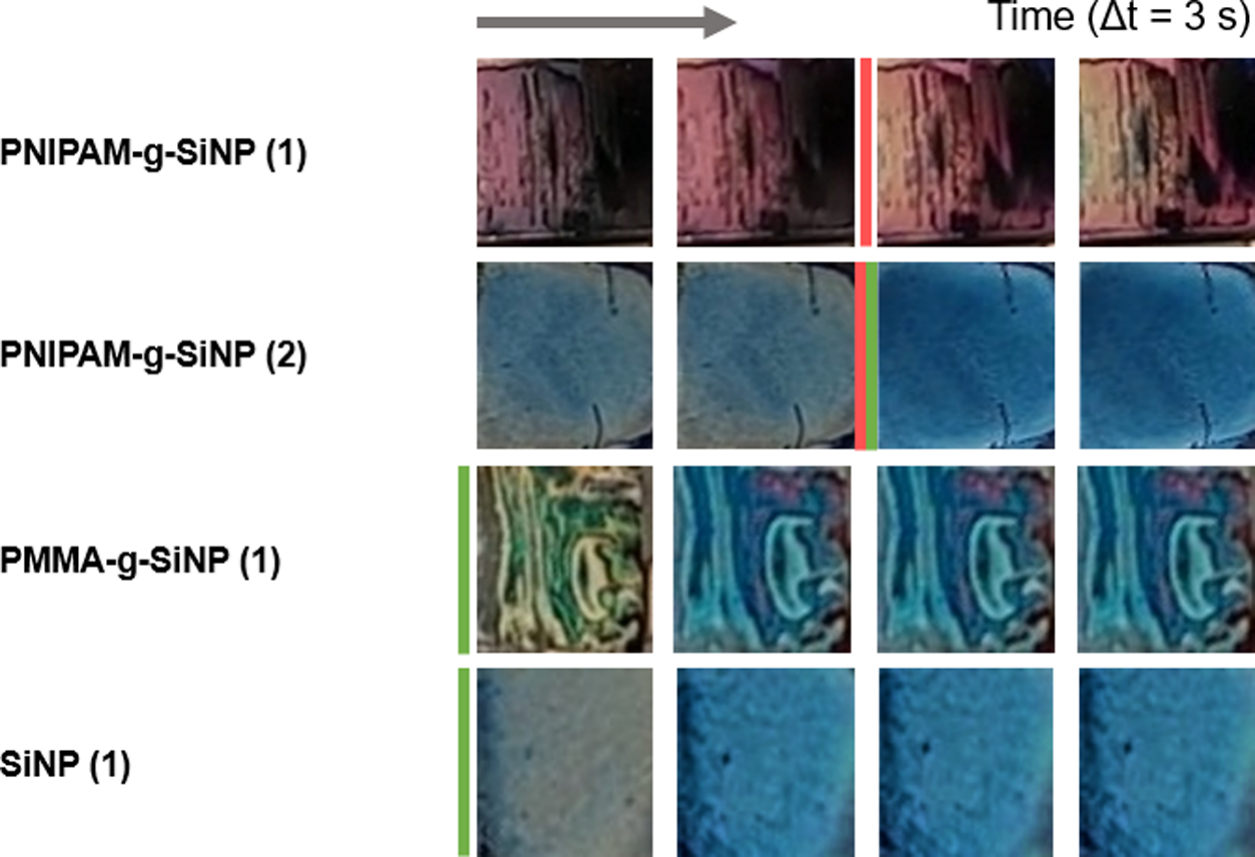
Images showing the first 12 s of the recovery phase in intervals of 3 s. Four different materials are considered and indicated per image series. A color change for PNIPAM-*g*-SiNP (1) is initiated from *t* ∼ 9 s (red), with a total recovery time of ∼36 s. PNIPAM-*g*-SiNP (2) shows a complete blue shift after *t* ∼ 6 s (green), and the reference materials of SiNP (1) and PMMA-*g*-SiNP (1) are already fully recovered at *t* < 3 s.

Other results from Table 1 reveal that thinner films of PNIPAM-*g*-SiNPs(2) display a similar delay in recovery of ∼6.3 s, followed by an immediate transition to their original color. Multilayered films of both reference materials also showed a color transition, which occurred much faster than the PNIPAM-*g*-SiNP films (0.4–1.5 s). The difference in color transitions is visualized in Figure 6, which shows screen captures of the films in the recovery phase. Screen captures with a time interval of Δ*t* of 3 s clearly show a different behavior between PNIPAM-*g*-SiNP films and the reference material. PNIPAM-*g*-SiNP(1) began to show a slight color change at *t* = 9 s, indicating the delay time. PNIPAM-*g*-SiNP(2), on the other hand, completely reverted to its original blue color in air at *t* = 9 s. For both PMMA-*g*-SiNP (1) and SiNP (1) materials, the color transition already occurred between *t* = 0 and *t* = 3 s. Given the fact that SiNP films will not experience an increase in nanostructural dimensions due to polymer brush swelling and that PMMA polymer brushes have a low affinity to ethanol,^43^ the results suggest that there are two effects influencing the stimuli-responsive behavior of stacked nanoparticle films. In the case of PNIPAM-*g*-SiNP materials, one influencing factor could be the swelling of PNIPAM brushes in ethanol vapor, hereby influencing *n*_eff_ and *D* parameters in eq 1. To test this hypothesis, AFM was used as a tool to monitor the structural dimensions of the nanoparticle films.

**Figure 7:**
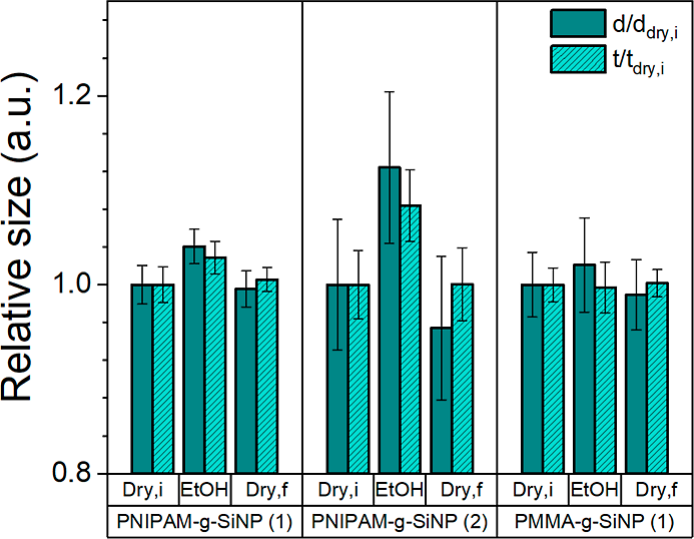
AFM data comparing the relative film thickness *t*/*t*_dry,i_ and relative nanoparticle diameter *d*/*d*_dry,i_ before, during, and after exposure to near-saturated ethanol vapor. The nanostructural dimensions and error bars were calculated based on multiple (*n* = 3–5) AFM images per state.

Figure 7 shows the relative size extracted from PNIPAM-*g*-SiNP and PMMA-*g*-SiNP films before, during, and after exposure to near-saturated ethanol vapor. This is referred to as the initial dry (dry,i), ethanol vapor (EtOH), and final dry (dry,f), respectively. From the acquired AFM images, the thickness of film *t* was compared to its original dry state *t*_dry,i_. The relative thickness, *t*/*t*_dry,i_, is an intercomparable measure for dimension changes in the *Z*-direction regardless of the initial film thickness *t*_dry,i_. Likewise, the relative nanoparticle diameter *d*/*d*_dry,i_ provides an intercomparable measure for diameter changes in the *XY*-direction regardless of the *d*_dry,i_ value.

By comparison of the two PNIPAM-*g*-SiNP materials with a PMMA-*g*-SiNP reference, the effect of ethanol vapor on the nanoparticle diameter becomes apparent. For the PNIPAM-*g*-SiNP (1) sample, an increase in the relative film thickness of 2.8 ± 1.5% and relative nanoparticle diameter of 4.0 ± 1.8% was observed. For the PNIPAM-*g*-SiNP (2) sample, relative dimensional changes of 8.4 ± 3.7 and 12.4 ± 8.0% were observed in ethanol vapor. These results suggest an absolute increase in PNIPAM brush height of ∼5–15 nm, with thicker films of PNIPAM-*g*-SiNPs showing a smaller increase in the nanoparticle diameter compared to thinner films of PNIPAM-*g*-SiNPs. We attribute this difference to the high spatial constraints for PNIPAM brush swelling in the multilayered nanoparticle films. From Figure 7, reversible swelling of the PNIPAM-*g*-SiNP materials is also evident by the decrease of the relative thickness and nanoparticle diameter after ethanol vapor exposure.

Ellipsometry measurements of a PNIPAM brush on a silicon substrate were done in saturated ethanol vapor to determine an indicative swelling ratio for the PNIPAM-*g*-SiNP materials (see Figure S7). With the obtained swelling ratio of α = 2.23 ± 0.13 and PNIPAM brush dry heights of 10–50 nm, the PNIPAM-*g*-SiNP materials were expected to swell >20 nm in the presence of saturated ethanol vapor. The reduced swelling capability of the PNIPAM-*g*-SiNP is likely due to spatial constraints in the nanoporous and closely packed films in all three dimensions.

As expected, the reference sample PMMA-*g*-SiNP (1) did not show a significant change during ethanol vapor exposure, with *t*/*t*_dry,i_ = 2.1 ± 3.8% and *d*/*d*_dry,i_ = −0.3 ± 2.6%. This is in line with the knowledge that PMMA brushes possess lower swelling ratios than PNIPAM brushes in ethanol media.^34,41,43^

With these results, it is established that a part of the stimuli-responsive behavior of stacked PNIPAM-*g*-SiNP films occurs due to swelling of the polymer brushes. Our measurements were conducted at room temperature (20 °C), which is below the lower-critical solution temperature (LCST) of PNIPAM of 32 °C.^41^ This means the polymer brushes can be assumed to be well-solvated^34^ and thus susceptible to ethanol vapor uptake. The influence of humidity on swelling of PNIPAM and PMMA brushes is expected to be negligible, as the relative humidity (RH) of ambient air in the laboratory facilities was measured at 30–40 RH%, and PNIPAM films show little swelling (α ∼ 1.02) at 40 RH%.^54^ At higher RH% values (>45 RH%), the influence of humidity on the material’s sensitivity to ethanol vapor cannot be neglected.^54,55^ In those cases, we suggest operating our sensor material above the LCST or developing polymer brush-grafted nanoparticle films with hydrophobic brushes. To the best of our knowledge, the extent of PNIPAM collapse in ethanol–water vapor mixtures above the LCST has not been investigated yet. In other words, the effectiveness of operating above the LCST to achieve high ethanol vapor selectivity toward humid air remains to be investigated.

At room temperature, we have now established that the PNIPAM brushes act as an absorber for ethanol vapor by which the material is able to maintain its color state longer. This is in line with the relatively long response/recovery times reported in polymer brush systems with acetone,^36^ methanol, and ethanol vapor stimuli.^39^ Delaying the color recovery with polymer brush coatings opens the possibility for ex situ sensing with structural colors.

The response of all multilayered nanoparticle films in ethanol vapor, regardless of their surface functionality, is thought to be due to condensation of ethanol vapor between the nanoparticles. Even small layers of condensed ethanol liquid will undoubtedly affect the effective refractive index *n*_eff_ in Bragg–Snell’s law and cause a red-shift color transition to occur. Ethanol is a volatile compound and evaporates easily, which means relaxation of the material to its original color should happen relatively fast and within seconds. Our assumption is consistent with the observations shown in Table 1, which indicate short relaxation times and no delay period for multilayered, nonfunctionalized SiNP films.

The relatively long recovery times of the multilayered PNIPAM-*g*-SiNP films could be explained by the influence of both ethanol vapor condensation and absorption in the PNIPAM brushes. The additional factor of vapor absorption in the PNIPAM brushes has been proven by AFM experiments and is shown in Figure 7. While condensed ethanol evaporates quickly, it is known that absorbed volatile compounds take relatively long to leave a polymer brush.^36^ Therefore, absorption of ethanol appears to be the reason for the delay period that PNIPAM-*g*-SiNP films consistently show in the recovery phase, regardless of the film thickness. While the two main contributing factors for vapor-responsive behavior in our structurally colored materials were identified, more research is needed to investigate the relative effects of vapor condensation and absorption in core–shell nanoparticle films. To extend our findings to other vapor-responsive systems, the minimum affinity between polymer chains and volatile analytes to achieve long recovery times must be examined. Next, to validate the sensor sensitivity, we suggest a follow-up study to assess the selectivity of our structurally colored materials with other VOCs. Improvements in this selectivity may be achieved by incorporating block-copolymer brushes or using multiple sensing platforms with different polymer brush-grafted nanoparticles.

## 4 Conclusions

This article shows a successful method to obtain ethanol vapor-responsive structural colors based on stacked PNIPAM-*g*-SiNPs. PNIPAM-*g*-SiNPs films of varying thicknesses and fabrication methods change color reversibly in near-saturated ethanol vapor. Herein, multilayered PNIPAM-*g*-SiNP films show delayed recovery characteristics, which occur in two phases: a sharply defined blue shift (delay) after 5.4–6.3 s and a gradual blue shift until a total recovery time of 33.3–36.2 s is reached (relaxation). Structural colors with nonfunctionalized SiNPs or PMMA-*g*-SiNPs also show a red-shift in an ethanol vapor environment but transit back to their original state 0.4–1.5 s after being exposed to air. With AFM, we validate that selective swelling of PNIPAM brushes takes place, which effectively alters the internal structure of the nanoparticle films and thus the structural color. The relatively long recovery times of our PNIPAM-*g*-SiNP films distinguish them from other vapor-sensitive structural colors and render the material highly suitable for ex situ vapor sensing.

## Supporting Information Available

Additional experimental data regarding the SiNPs, structural colors, and their vapor sensing characteristics (PDF)
