# Supplementary material for: PNIPAM Brushes in Colloidal Photonic Crystals Enable Ex Situ Ethanol Vapor Sensing
Source: ACS Appl Polym Mater. 2023 Dec 14;6(1):870–8. doi: 10.1021/acsapm.3c02397 (PMC10788857; doi:10.1021/acsapm.3c02397)
Supplement: Supplementary file 1 — ap3c02397_si_001.pdf [file ap3c02397_si_001.pdf]

Electronic Supplementary Information

PNIPAM Brushes in Colloidal Photonic Crystals Enable Ex Situ Ethanol Vapor Sensing

Esli Diepenbroek, Maria Brió Pérez, Sissi de Beer*
Department of Molecules & Materials, MESA+ Institute, University of Twente, 7522 NB Enschede, the Netherlands
* Email: s.j.a.debeer@utwente.nl

Synthesis of polymer brush-grafted nanoparticles

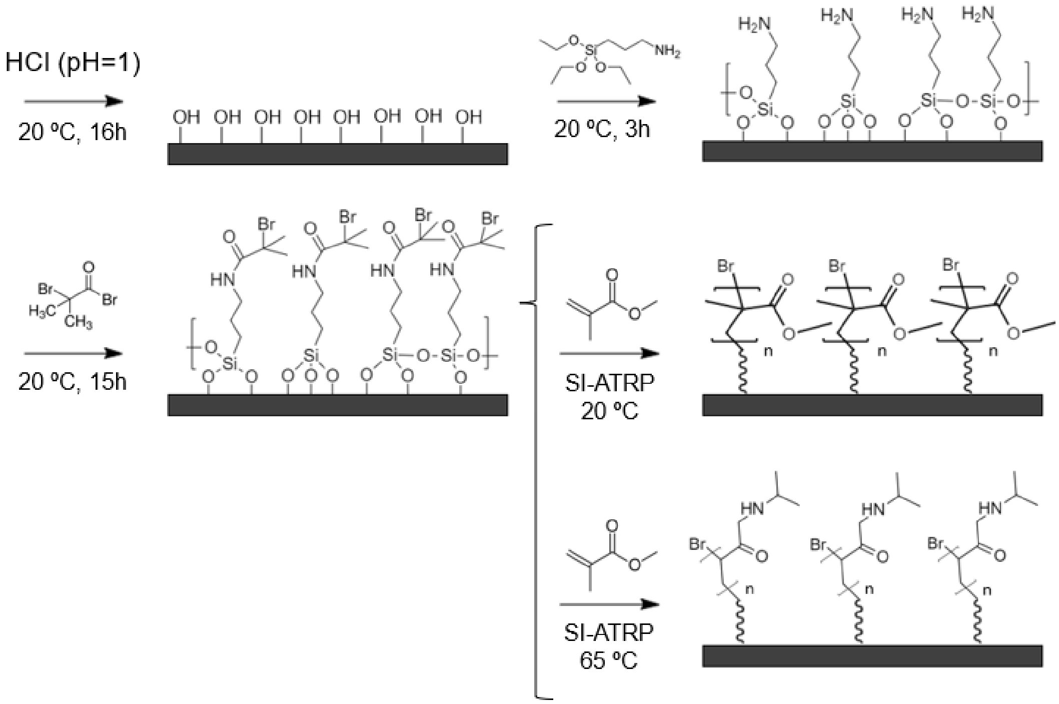

Scheme S1 Reaction scheme of the SiNP surface functionalization steps to obtain PNIPAM-g-SiNPs and PMMA-g-SiNPs. In the first step, the silica surface is hydrolyzed by HCl (pH = 1) for 16h at 20 °C. The OH-moieties subsequently react with APTES for 3h at 20 °C to form an anchoring layer. The NH_2-moieties of APTES react with one bromide group of BiBB in the next step, yielding a Br-functionalized nanoparticle surface after a 15h reaction (20 °C). The SI-ATRP reactions with MMA (row 2) and NIPAM monomers (row 3) proceed with different reaction times to obtain polymer brushes of varying thicknesses.

Characterization of polymer brush-grafted nanoparticles

PNIPAM-g-SiNPs and PMMA-g-SiNPs were characterized by SEM, DLS, FTIR and TEM to proof the successfulness of the Stöber reaction and nanoparticle surface functionalization. Moreover, the acquired data were used to determine the PNIPAM and PMMA brush thicknesses and swelling characteristics. The following section contains figures with our non-processed SEM and DLS data (Figure S1), TEM images of PNIPAM-g-SiNPs (Figure S2), FTIR spectra of functionalized SiNPs (Figure S3) and AFM images of PNIPAM-g-SiNP films (Figure S4). Table S1 contain results on polymer brush thicknesses.

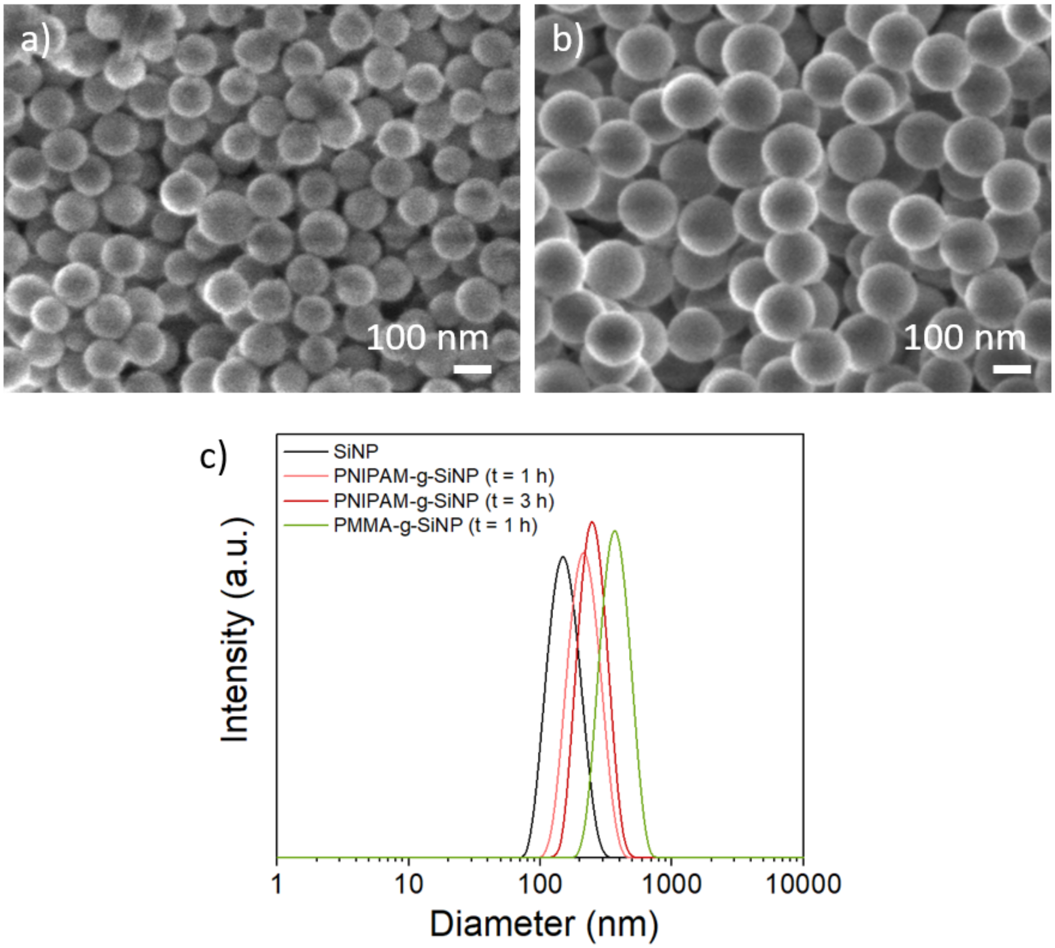

Figure S1 a) SEM image of PNIPAM-g-SiNPs, obtained after a SI-ATRP reaction of $t = 1$ hour. The image is taken at 70,000x magnification and 1.5 kV acceleration voltage. b) SEM image of PMMA-g-SiNPs, obtained after a SI-ATRP reaction of $t = 1$ hour. The image is taken at 70,000x magnification and 1.5 kV acceleration voltage. c) DLS spectra of non-functionalized and functionalized SiNPs. To obtain stable dispersions, DLS samples of SiNPs and PNIPAM-g-SiNPs were prepared in ethanol solvent and PMMA-g-SiNPs in acetone solvent.

Table S1 Determination of the dry polymer thickness by SEM and TEM, using ImageJ image analysis software. The method, sample type, SI ATRP reaction time ($t_{\text{SI-ATRP}}$) and dry brush height (h_{dry}) are denoted for five different samples. The values of h_{dry} were obtained by averaging > 15 measurements per at least 2 SEM or TEM images.

**Characterization type**	**Sample**	$\mathbf{t_{SI\text{-}ATRP}}$ **(h)**	$\mathbf{h_{dry}}$ **(nm)**
SEM	PNIPAM-g-SiNP	1	30.4 ± 4.2
SEM	PNIPAM-g-SiNP	1	33.7 ± 8.4
SEM	PMMA-g-SiNP	1	57.3 ± 9.6
SEM	PMMA-g-SiNP	1	62.8 ± 15.2
TEM	PNIPAM-g-SiNP	0.5	7.9 ± 1.1

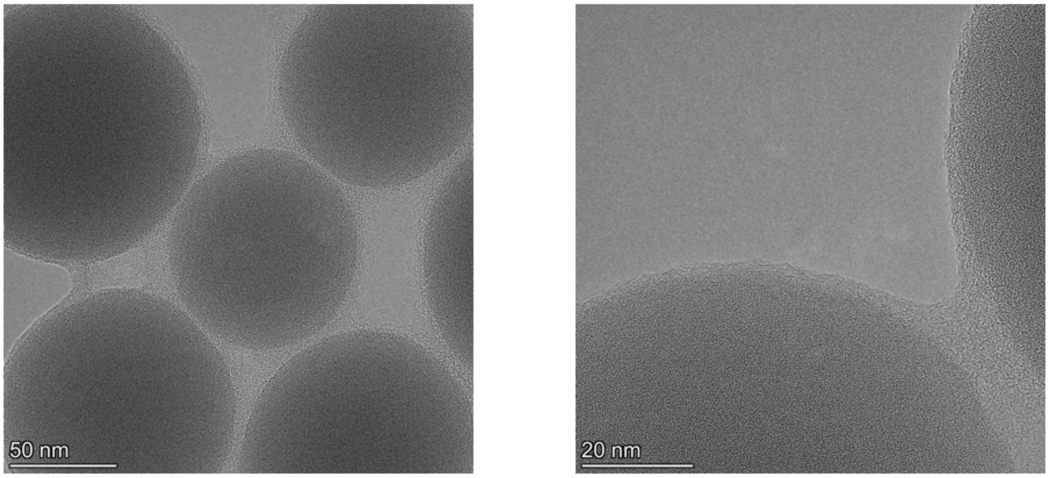

Figure S2 TEM images of PNIPAM-g-SiNPs magnified at 145.000x (left) and 380.000x (right). With a SI-ATRP reaction time of $t = 0.5$ hour, a small polymeric shell of PNIPAM can be recognized.

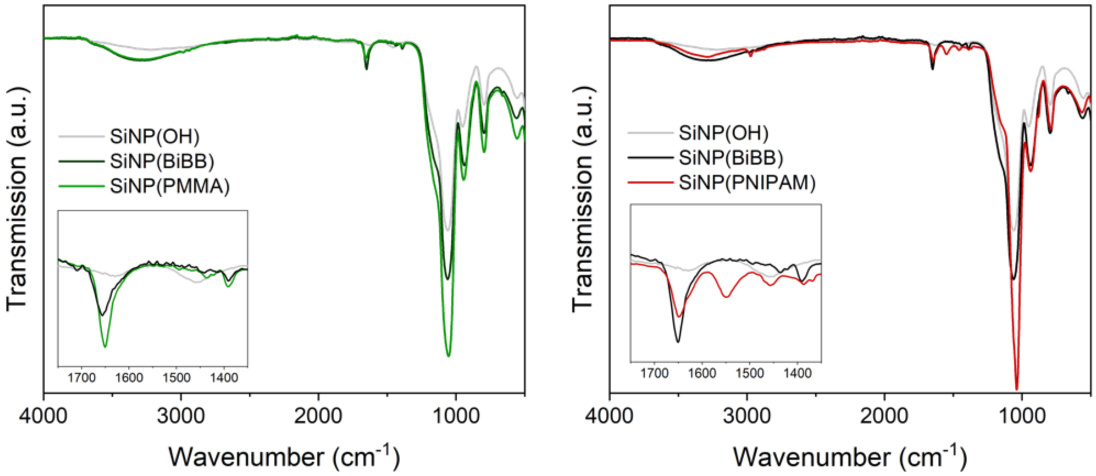

Figure S3 FTIR spectra of the PMMA-grafted nanoparticles (left) and the PNIPAM-grafted nanoparticles (right). The bare and BiBB-functionalized nanoparticles are shown in grey and black, respectively, to mark differences between the FTIR peaks. Relevant peaks at 1350-1750 cm^{-1} are added in a separate zoom-in.

Ethanol vapor-responsive characteristics

The swelling characteristics of PNIPAM-g-SiNPs and PMMA-g-SiNPs in ethanol vapor were determined via DLS/SEM and AFM. In addition, ellipsometry measurements were performed on PNIPAM and PMMA brushes grafted onto SiO_2 wafers. From the DLS/SEM and ellipsometry results, the swelling ratio α could be calculated via the following equation:

$$\alpha = \frac{h_{swell}}{h_{dry}} \tag{1}$$

with h_{swell} being the swollen height of a polymer brush in nm, and h_{dry} being the corresponding dry height in nm. In the DLS/SEM methodology h_{swell} is extracted from DLS data, whereby the nanoparticles are being dissolved in a solvent with a high affinity to the polymer brush in order to avoid aggregation. The h_{dry} value is extracted from SEM, as was previously shown in Table S2.

The following section contains an overview of the swelling characteristics of PNIPAM and PMMA brushes (Table S2), AFM images from the structurally colored material studies in ethanol vapor (Figure S4), a comparison study between the Bragg-Snell's theorem and observed coloration (Figure S5), a parameter influence study for the prediction of the reflected wavelength (Table S3), a qualitative material study of the sensitivity towards ethanol vapor (Figure S6), a data overview of the long-term optical stability (Table S4) and ellipsometry data obtained during a swelling experiment in ethanol vapor (Figure S7).

Table S2 Swelling characteristics of PNIPAM and PMMA brushes in different geometries. The swelling medium, characterization method and swelling ratio (α) were denoted for PNIPAM and PMMA brushes on SiNP or SiO_2 substrates. The difference between PNIPAM and PMMA in their affinity towards ethanol as a swelling medium is noticeable by the difference in α values.

**Geometry**	**Brush type**	**Medium**	Method	α **(a.u.)**
SiNP ($d \sim$ 125.5 nm)	PNIPAM	Ethanol (solvent)	DLS	3.06 ± 0.54
SiNP ($d \sim$ 125.5 nm)	PMMA	Acetone (solvent)	DLS	3.48 ± 0.67
SiO_2 wafer (6x2 cm^2)	PNIPAM	Ethanol (solvent)	Ellipsometry	3.71 ± 0.44
SiO_2 wafer (6x2 cm^2)	PNIPAM	Ethanol (vapor)	Ellipsometry	2.23 ± 0.13
SiO_2 wafer (6x2 cm^2)	PMMA	Ethanol (solvent)	Ellipsometry	1.60 ± 0.29

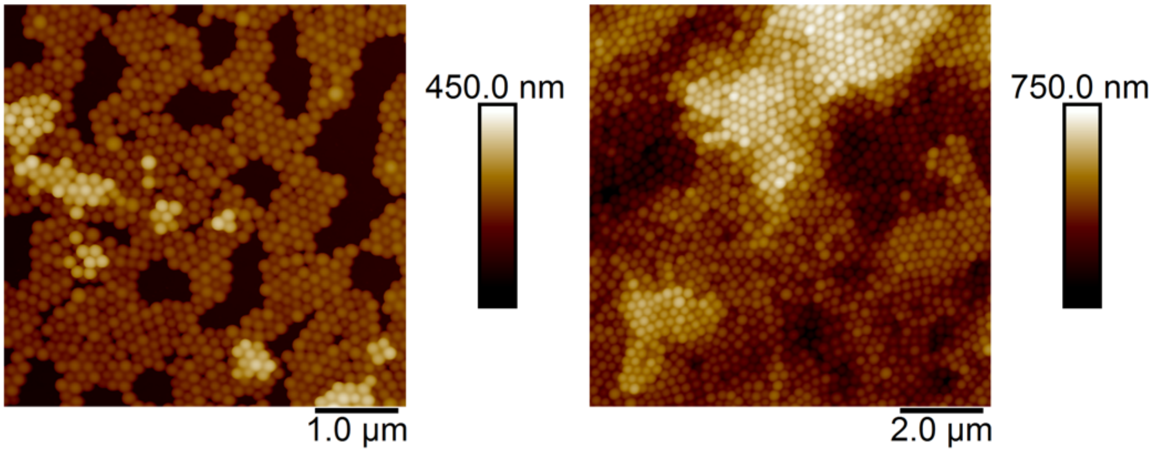

Figure S4 Top surface AFM images of PNIPAM-g-SiNP films before exposure to near-saturated ethanol vapor. The tapping mode in air was used to image a thin dipcoated film (PNIPAM-g-SiNP(2), left) and a thick self-assembled film (PNIPAM-g-SiNP(1), right). The mono- to bi-layer formation is clearly visible in the left PNIPAM-g-SiNP(2) material, with the nanoparticle closely packed in a FCC(100) ordering. The PNIPAM-g-SiNP(1) sample shows an identical close packing of the nanoparticles, with terrace formation on the micrometer scale.

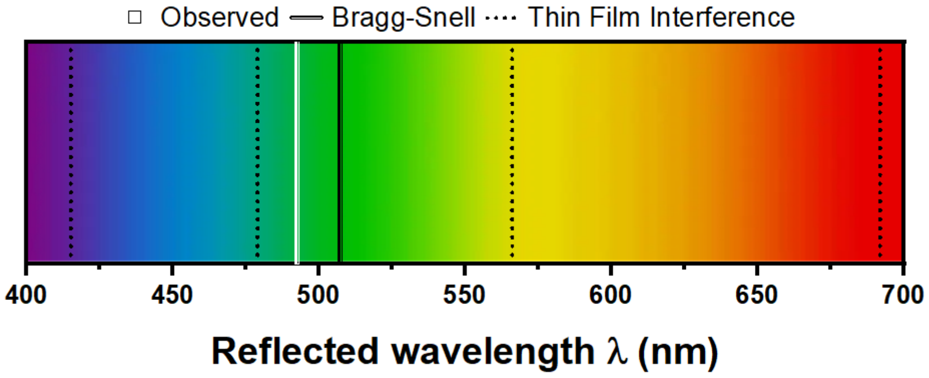

Figure S5 Comparison study to verify the origin of structural coloration of our materials. The observed reflection peak (white line) is put next to the expected Bragg-Snell reflection (double black line) and reflection peaks due to thin film interference (dotted black lines). Thin film interference values were calculated via $\lambda = \frac{2\, n_{\text{eff}} t}{m+0.5}$ Assumed is $n_{\text{eff}} = 1.39$, $D = 223.5$ nm, $t = 1152$ nm, and $\theta = 0^{\circ}$. From this study, it is clear that the materials can be considered a colloidal photonic crystal rather than a thin film.

Table S3 Parameter influence study of colloidal spacing D and effective refractive index n_{eff} on the calculation of the reflected wavelength λ_{calc} via Bragg-Snell's law. The pre-determined variable is $\theta_0 = 0\,^{\circ}$.

D (nm)	n_{eff} (a.u.)	λ_{calc} (nm)
223.5	1.39	507.3
220.0	1.39	499.4
230.0	1.39	522.1
223.5	1.35	492.7
223.5	1.43	521.9

For the comparison study between Bragg-Snell law's theoretical predictions and our experimental findings, we estimate the effective refractive index n_{eff}. Our structural colors consist of three different materials for vapor sensing, which are Stöber silica (nanoparticles), PNIPAM (brushes) and air. The bulk refractive indices are 1.475,[1] 1.5031[2] and 1.0003,[3] respectively. A modified Lorentz-Lorentz relation is used to estimate the value of n_{eff}:

$$\frac{n^2{}_{\text{eff}}-1}{n^2{}_{\text{eff}}+2} = f_{\text{SiO}_2}\frac{n^2{}_{\text{SiO}_2}-1}{n^2{}_{\text{SiO}_2}+2} + f_{\text{PNIPAM}}\frac{n^2{}_{\text{PNIPAM}}-1}{n^2{}_{\text{PNIPAM}}+2} + f_{\text{air}}\frac{n^2{}_{\text{air}}-1}{n^2{}_{\text{air}}+2} \tag{2}$$

where f denotes the respective volume fractions of air, SiO_2 and PNIPAM. In our calculations for the effective refractive index, we assume a FCC colloidal packing. Our assumption is supported by the AFM images in Figure S4, which show a tight packing of the core-shell nanoparticles. For a FCC packing, the volume fraction f_{air} is equal to 0.26.[4] The core-shell particles, consisting of a SiO_2 core and PNIPAM shell, are thus assumed to take up a total of 74% of the material's volume. The corresponding volume fractions for SiO_2 and PNIPAM are predicted on the basis of Table S1 and the following formula:

$$f_{\text{SiO}_2} = \frac{d_{\text{SiO}_2}{}^3}{\left(d_{\text{SiO}_2} + 2h_{\text{dry}}\right)^3} \tag{3}$$

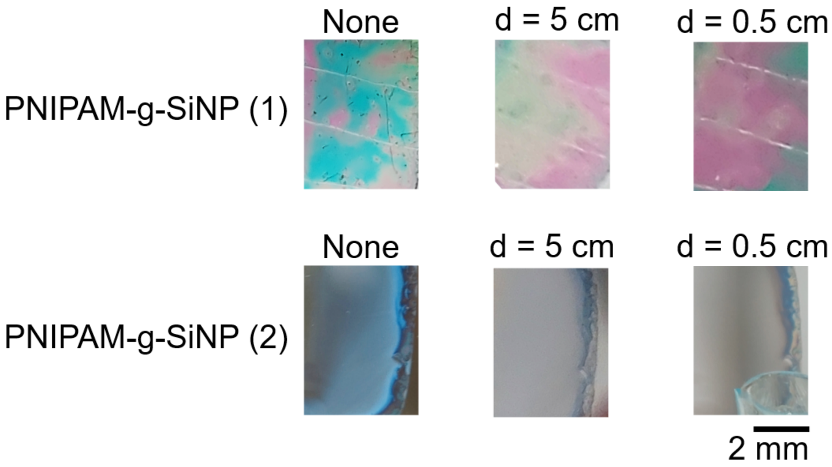

Figure S6 Qualitative sensitivity study of PNIPAM-g-SiNP(1) and PNIPAM-g-SiNP(2) films towards ethanol vapor. A gentle ethanol vapor flow was positioned d = 5 cm and d = 0.5 cm from the structurally color samples to mimic a different ethanol vapor concentration. The original color without ethanol vapor exposure is depicted on the left pictures. In this comparison, it can be seen that the thick PNIPAM-g-SiNP(1) films show a gradual red-shift color transition for decreasing exposure distances. PNIPAM-g-SiNP(2) film do not show this gradual color transition.

Table S4 Optical stability of a PNIPAM-g-SiNP structural color over time. The material properties, including the delay time, relaxation time, reflection peak in air ($\lambda_{max,air}$) and reflection peak in saturated ethanol vapor ($\lambda_{max,EtOH}$) were measured fresh (*t = 0 days*) and after a period of ~8 months (*t = 250 days*).

	**t = 0 days**	**t = 250 days**
Delay (s)	5.4 ± 2.8	5.7 ± 1.9
Relaxation (s)	34.0 ± 7.1	42.0 ± 8.4
$\lambda_{max,air}$ (nm)	661 ± 2	664 ± 4
$\lambda_{max,EtOH}$ (nm)	447 ± 3	447 ± 18

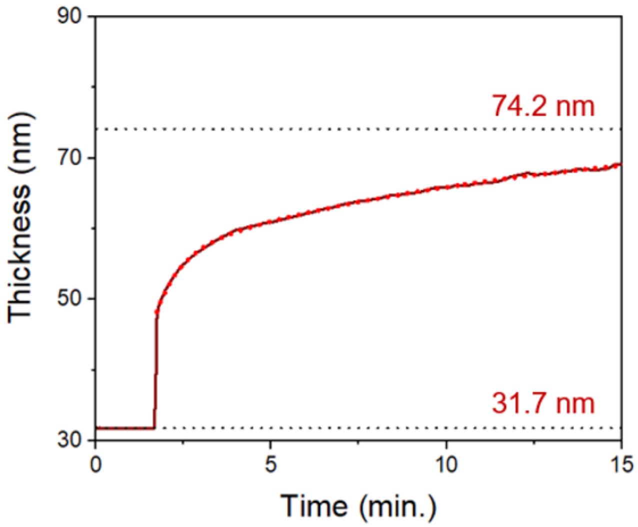

Figure S7 Swelling response of a PNIPAM polymer brush in saturated ethanol vapor (flow: 800 ml/min), as measured by ellipsometry. The flow is switched between 800 ml/min N_2 to ethanol vapor at $t \sim 2$ minutes. The thickness of the brush is determined via a Cauchy optical model and the swelling ratio is determined by considering h_{swell} = 69.5 nm and h_{dry} = 31.7 nm and using eq. 1. The equilibrated swollen height h_{swell} was determined via an exponential fit, indicated by the dotted red line.

References

[1] Boris N. Khlebtsov, Vitaly A. Khanadeev, and Nikolai G. Khlebtsov. Determination of the size, concentration, and refractive index of silica nanoparticles from turbidity spectra. *Langmuir*, 24: 8964—-8970, 2008. doi: 10.1021/la8010053.

[2] Yannic Brasse, Mareen B. Müller, Matthias Karg, Christian Kuttner, Tobias A.F. König, and Andreas Fery. Magnetic and Electric Resonances in Particle-to-Film-Coupled Functional Nanostructures. *ACS Appl. Mater. Interfaces*, 10:3133–3141, 2018. doi: 10.1021/acsami.7b16941.

[3] Philip E. Ciddor. Refractive index of air: new equations for the visible and near infrared. *Appl. Opt.*, 35:1566–1573, 1996. doi: 10.1364/AO.35.001566.

[4] Wilson C. K. Poon, Eric R. Weeks, and C. Patrick Royall. On measuring colloidal volume fractions. *Soft Matter*, 8:21–30, 2012. doi: 10.1039/C1SM06083J.
